# Identification, Bioaccessibility, and Antioxidant Properties of Phenolic Compounds in Carob Syrup

**DOI:** 10.3390/foods13142196

**Published:** 2024-07-11

**Authors:** Melissa Zannini, Alice Cattivelli, Lorenzo Nissen, Angela Conte, Andrea Gianotti, Davide Tagliazucchi

**Affiliations:** 1 Nutritional Biochemistry Lab, Department of Life Sciences, University of Modena and Reggio Emilia, Via Amendola 2, 42122 Reggio Emilia, Italy; melissa.zannini@unimore.it (M.Z.); alice.cattivelli@unimore.it (A.C.); davide.tagliazucchi@unimore.it (D.T.); 2 Department of Agricultural and Food Sciences (DISTAL), Alma Mater Studiorum—University of Bologna, Piazza Goidanich 60, 47521 Cesena, Italy; lorenzo.nissen@unibo.it (L.N.); andrea.gianotti@unibo.it (A.G.); 3 Interdepartmental Centre of Agri-Food Industrial Research (CIRI), Alma Mater Studiorum—University of Bologna, Piazza G. Goidanich, 47521 Cesena, Italy; 4 Centre for Applied Biomedical Research—CRBA, Alma Mater Studiorum—University of Bologna, Policlinico di Sant’Orsola, Via Massarenti 9, 40138 Bologna, Italy

**Keywords:** mass spectrometry, *Ceratonia siliqua*, polyphenols, anti-oxidant activity, bioactive compounds

## Abstract

Carob syrup is a brown, thick syrup produced from carob pulp that can be directly consumed or used as a sweetener, which also finds applications in folk medicinal practices. In this work, the quali–quantitative phenolic profile of five different carob syrups was elucidated before and after in vitro gastro–intestinal digestion. Moreover, the anti-oxidant properties of undigested and digested carob syrups were investigated. A total of 75 phenolic compounds were identified in undigested carob syrups. The most important phenolic compound in all the samples was gallic acid, the concentration of which ranged between 54.28 and 117.73 mg/100 g. Additional compounds belonging to the classes of hydroxybenzoic acids (in particular glycosylated gallic acid derivatives), hydroxycinnamic acids, and flavonoids (especially flavonols) were also identified. During in vitro gastric digestion, gallic acid mono- and di-hexosides were diglycosylated, releasing gallic acid, which was further degraded in ellagic acid through oxidative polymerization in the intestinal phase of the digestion. Ellagic acid was the major compound detected after in vitro gastro–intestinal digestion of carob syrups. With few exceptions, the anti-oxidant properties of carob syrup were preserved even after digestion. Carob syrup can be considered an important source of phenolic compounds with demonstrated positive effects on human health.

## 1. Introduction

The carob tree (*Ceratonia siliqua* L.) is an evergreen plant belonging to the Fabaceae family, notably widespread in the Mediterranean area, especially in European countries such as Spain, Italy, Greece, Cyprus, and Portugal as well as in Turkey, Siria, and Morocco. The carob tree fruits are composed of an external pod that includes a fleshy pulp that incorporates the seeds [1].

Both carob pulp and seeds have been widely used as raw materials in food industries to develop food ingredients and products. Carob seeds are mainly used to produce locust bean gum used as a food thickener and stabilizer [2]. Furthermore, carob pulp can be processed to obtain several human foods, such as carob syrup, carob powder (or carob flour), and carob fiber (de-sugared carob powder) [1,3]. The main application of carob powder is as a cocoa substitute, whereas carob syrup is predominantly used as a sweetener [1,3,4].

Among the different carob products, one of the most popular and marketable is carob syrup [4]. Carob syrup is produced from grounded carob pulp by solid-liquid extraction with boiling water following concentration at a high temperature to obtain a brown product with a sugar content higher than 60% [5]. Its principal use is as a sweetener in the making of cookies, cakes, or other desserts. For its high sugar content, it is also utilized for softening and conserving fruits. In addition, in folk medicinal practices, carob syrup is also used in treating coughs, sore throats, and gastro–intestinal disorders.

Recently carob products, including carob syrup, have received great attention in the light of their potential health benefits [1,3,4]. For example, in vivo studies on rats demonstrated that carob syrup administration resulted in lipid-lowering effects, decreasing both total triglycerides and cholesterol and increasing HDL levels [6]. In addition, carob syrup exhibited in vitro high anti-oxidant, anti-inflammatory, and anti-proliferative activities [7,8,9]. Notably, some of the health benefits of carob syrup are related to the gastro–intestinal tract. In particular, carob syrup decreased the proliferation of the colon adenocarcinoma cell line by promoting apoptosis [10]. Furthermore, carob syrup displayed anti-diarrheal activity mainly thanks to its ability to inhibit the growth of pathogenic bacteria (such as *Escherichia coli*) [8,11]. 

Most of the biological effects of carob extracts and products have been related to the presence of phenolic compounds [3]. Several phenolic compounds belonging to different classes have been identified and quantified in carob products. The most prominent class of phenolic compounds in carob was represented by phenolic acids (mainly gallic acid, protocatechuic acid, coumaric acid, ferulic acid, and caffeic acid), gallotannins, flavanols (catechins and procyanidins) and flavonols (quercetin- and myricetin-derivatives) [8,12,13]. The quali–quantitative phenolic profile differed depending on the carob products. For example, in carob fiber, the most important phenolic compounds were flavonols and hydrolyzable gallotannins, whereas, in carob syrup, the phenolic profile was dominated by gallic acid that may represent up to 90% of total phenolic compounds [8,9,13].

To exert their effects, phenolic compounds must be liberated from the food matrix and stable during gastro–intestinal digestion, i.e., they must be bio-accessible [14,15]. Phenolic compound bioaccessibility is essentially dependent on the chemical characteristics of the molecules, the food matrices, and the gastro–intestinal tract environment (such as pH, digestive enzymes, bile salts, etc.) [16,17,18]. In vitro gastro–intestinal digestion models have been widely used to study the release and stability of phenolic compounds from vegetable foods [17,18]. In particular, the development of the INFOGEST protocol that closely mimics the physiological conditions made it possible to harmonize the data obtained from the various laboratories, making them more reproducible and predicting what could happen in vivo [18].

Even so, to the best of our knowledge, there is only limited information on the phenolic profile of carob syrup. Indeed, no studies have been carried out to understand the fate of carob syrup phenolic compounds during in vitro gastro–intestinal digestion.

To fill this gap, the present work aims to give a detailed and comprehensive picture of the phenolic profile of different carob syrups by high-resolution mass spectrometry. Next, the stability and release from the food matrix (i.e., the bioaccessibility) of carob syrup phenolic compounds were assessed after in vitro gastro–intestinal digestion. Finally, the anti-oxidant properties of undigested and digested carob syrups were evaluated by various assays.

## 2. Materials and Methods

### 2.1. Samples and Materials 

Enzymes (human salivary α-amylase, porcine pepsin, and porcine pancreatin) and chemicals for in vitro gastro–intestinal digestion and anti-oxidant activity assays were obtained from Sigma (Milan, Italy), whereas the solvents (MS grade) for high-resolution mass spectrometry and phenolic compounds extraction were purchased from BioRad (Hercules, CA, USA). Standard phenolic compounds used for MS quantification are reported in Table S1. Five samples of carob syrup were obtained from different suppliers and indicated as follows: C, L, S1, S2, and S3. Products C (Mavroudes, Cyprus) and L (Loman, Italy) were purchased by e-commerce and stored at room temperature before analyses conducted much time before their commercial shelf life or expiration date. Products S1, S2, and S3 are three Tunisian artisanal products produced in the Teboulba region, Bekalta region, and Moknine region, which are included in the European PRIMA Project GourMed 2021–2024 (www.gourmed-prima.com).

### 2.2. Phenolic Compounds Extraction from Carob Syrups

Extraction of carob syrup phenolic compounds was carried out by mixing 0.5 g of carob syrup with 0.5 mL of methanol/water/formic acid solution (70:28:2, *v*/*v*). The mixtures were vortexed for 1 min and then subjected to centrifugation for 20 min (6000× *g*; 4 °C). Finally, the collected supernatants were stored at −20 °C until further analysis.

### 2.3. In Vitro Digestion of Carob Syrups

In vitro gastro–intestinal digestion was performed by applying the INFOGEST 2.0 protocol [19]. Briefly, 1 g of carob syrup was mixed with 1 mL of simulated salivary fluid containing 150 U/mL of salivary α-amylase. After 2 min of incubation at 37 °C in a rotating wheel (10 rpm), 2 mL of simulated gastric fluid was added, and the pH was corrected to 3 with HCl 6 mol/L. The gastric digestion was started by the addition of 2000 U/mL (final concentration in the digestive system) of pepsin (100 μL of pepsin dissolved in water), followed by 120 min of incubation at 37 °C in a rotating wheel (10 rpm). Next, the bolus was mixed with 4 mL of simulated intestinal fluid. The pH was brought to 7.5 with concentrated NaOH, and the mixture was equilibrated by 30 min of incubation at 37 °C in a rotating wheel (10 rpm). Intestinal digestion was started by adding to the chyme pancreatin (final concentration in the digestive system based on trypsin activity of 200 U/mL), followed by 120 min of incubation at 37 °C in a rotating wheel (10 rpm). A control digestion with water instead of carob syrup was performed to account for the possible interferences in the assays of the digestive system. At the end of the intestinal digestion, samples were centrifuged at 10,000× *g* for 20 min at 4 °C, and the supernatant was stored at −20 °C until analysis.

### 2.4. Identification and Quantification of Phenolic Compounds by High-Performance Mass Spectrometry in Chemical Extracts and In Vitro Digested Samples

The phenolic profiles of carob syrup extracts and in vitro digested samples were analyzed by high-resolution mass spectrometry using a Q Exactive Hybrid Quadrupole-Orbitrap Mass Spectrometer coupled to a UHPLC Ultimate 3000 module (Thermo Fisher Scientific, San Jose, CA, USA). Samples were directly injected in the MS instrument after centrifugation (10,000× *g*; 20 min; 4 °C), filtration with 0.22 μm syringe filter, and appropriate dilution. Phenolic compounds were separated with a C18 column (Acquity UPLC HSS C18 Reversed phase, 2.1 × 100 mm, 1.8 µm particle size, Waters, Milan, Italy). The applied gradient, the flow rate as well as the mass spectrometry setting are reported in Martini et al. [20]. Quantification was carried out by building external calibration curves with the available standard compounds, as depicted in Table S1. Data are expressed as mg/100 g of carob syrup. The bioaccessibility index (BI) was calculated for each individual phenolic compound as well as for the phenolic classes and total phenolic compounds, as previously reported [21].

### 2.5. Total Phenolic Compounds Quantification and Anti-Oxidant Activity Assays

#### 2.5.1. Total Phenolic Compounds Quantification

The total phenolic compounds in carob syrup extracts and digested samples were determined by the Folin–Ciocalteau assay as previously described [22]. The results were expressed as mg of gallic acid per 100 g of carob syrup.

#### 2.5.2. Assessment of the ABTS Radical Scavenging Activity

The radical scavenging ability of carob syrup extracts and digested samples was assessed using the ABTS assay, according to Re et al. [23]. The ABTS scavenging ability was reported as mg of Trolox equivalent per 100 g of carob syrup. 

#### 2.5.3. Determination of the Reducing Ability

The reducing ability of carob syrup extracts and digested samples was performed using the protocol based on the ferric-reducing/anti-oxidant power (FRAP) as described in Benzie and Strain [24]. FRAP data were reported as mg of FeSO_4_ equivalent per 100 g of carob syrup. 

#### 2.5.4. Evaluation of the Inhibitory Activity against Fenton Reaction

The capacity to inhibit the progression of the Fenton reaction was evaluated as previously reported [25]. Results were reported as μmol of ascorbic acid equivalent per 100 g of carob syrup.

### 2.6. Determination of Browning Index

The Browning index of carob syrups was determined by measuring the specific extinction coefficient at 420 nm (Kmix 420 nm) of the carob syrups. The Kmix 420 nm value is defined as the absorption at 420 nm of carob syrup solution at the concentration of 1 g/L and expressed in L/g×m [26]. For the spectroscopic measurement, a solution of carob syrup at a concentration of 1 mg/mL was prepared by diluting the original carob syrup in water and read at 420 nm with a spectrophotometer.

### 2.7. Statistics

Data are reported as mean ± SD for three analytical replicates for each analyzed sample. Phenolic extraction and in vitro gastro–intestinal digestion were performed in triplicate for each sample. Differences between samples were established by one-way univariate analysis of variance (ANOVA) with Tukey’s post hoc test using Graph Pad Prism 6.0 (GraphPad Software, San Diego, CA, USA). The differences were considered significant with *p* < 0.05. Pearson correlation analysis was carried out using the software MetaboAnalyst 5.0 [27].

## 3. Result and Discussion

### 3.1. Total Phenolic Compounds, Anti-Oxidant Properties and Browning Index of Carob Syrups

**Figure 1:**
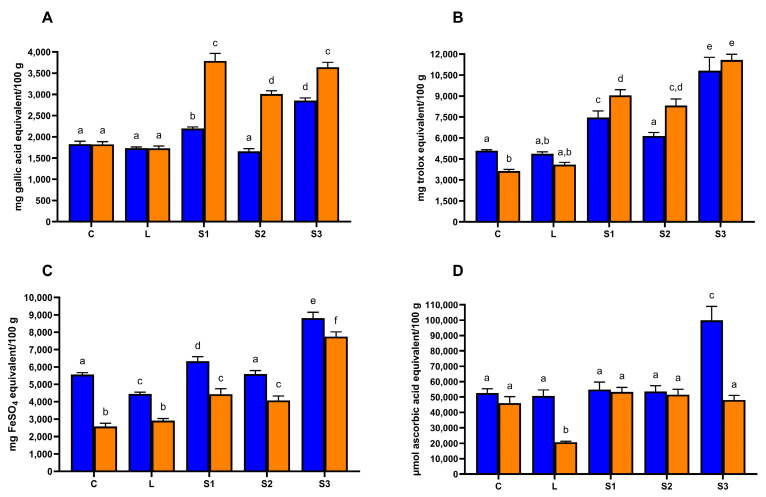
Total phenolic compounds and anti-oxidant properties of carob syrups before (blue bars) and after (orange bars) in vitro gastro–intestinal digestion. (**A**) Total phenolic compounds determined by the Folin–Ciocalteau assay. Results are expressed as mg of gallic acid equivalent/100 g of carob syrup. (**B**) ABTS radical scavenging activity. Results are expressed as mg of Trolox equivalent/100 g of carob syrup. (**C**) Ferric-reducing ability (FRAP assay). Results are expressed as mg of FeSO_4_ equivalent/100 g of carob syrup. (**D**) Fenton reaction inhibition. Results are expressed as μmol of ascorbic acid/100 g of carob syrup. Different letters mean significant differences (*p* < 0.05).

The total phenolic content of the different carob syrup samples, determined by the Folin–Ciocalteau assay, is shown in Figure 1A.

The highest total phenolic content was found in carob syrup S3 (2853.33 ± 62.92 mg of gallic acid equivalent/100 g of carob syrup), followed by sample S1 (2195.00 ± 38.19 mg of gallic acid equivalent/100 g of carob syrup). Significantly lower values (*p* < 0.05) of total phenolic compounds were found for samples C, L, and S2, ranging from 1658.89 ± 62.55 and 1828.33 ± 66.67 mg of gallic acid equivalent/100 g of carob syrup in samples S2 and C, respectively. Total phenolic compound amounts in these samples were not significantly different (*p* > 0.05). The total phenolic content determined in the present study in the carob syrups was higher than previously reported in the literature [8,9,13,28]. Differences in total phenolic compound data reported in the literature and among the carob syrups analyzed in the present study may be related to several factors, such as processing method, thermal treatment, fruit maturation, and geographical origin [8,29,30].

The anti-oxidant properties of carob syrups were ascertained by three different assays. The trend of the radical scavenging ability of carob syrups against the radical ABTS (Figure 1B) was similar to that of total phenolic compounds, with the samples S3 and S1 showing the highest scavenging capacity. The same conclusion can be reached for the ferric-reducing ability of carob syrup samples (Figure 1C). Previous studies found that carob syrup displayed a strong scavenging capacity against the radical ABTS and the ferric-reducing ability [8,9,28]. However, it is difficult to compare data in the literature due to the different standards used for the assays and the distinct ways of expressing the results (e.g., mg, μmol, or IC_50_). Finally, as reported in Figure 1D, all the carob syrup samples were able to inhibit the progression of the Fenton reaction. No significant differences (*p* > 0.05) were found among the samples C, L, S1, and S2, whereas carob syrup S3 displayed significantly higher (*p* < 0.05) activity. 

The production procedure of carob syrup, which involves boiling to concentrate the product, and the presence of high amounts of reducing sugars and amino acids/proteins promotes the Maillard reaction as indicated by the brown color of the samples. Non-enzymatic Browning reactions are often desirable since they may improve sensorial characteristics (such as flavor and aroma) of foods and lead to the production of bioactive compounds such as high-molecular-weight melanoidins with strong anti-oxidant activity [31]. However, the Maillard reaction may result in a depletion of some nutrients (e.g., amino acids) and other bioactive molecules, such as phenolic compounds, which can be incorporated into the melanoidin structure [32]. Moreover, thermal treatment may promote the degradation of phenolic compounds [29]. Therefore, as an indication of the melanoidin content and the extent of the thermal treatments, the Browning index was determined for the different carob syrup samples (Supplementary Table S2).

The highest value of Kmix 420 nm was found for carob syrup C (0.402 ± 0.02), followed by samples S1 and L (0.255 ± 0.01 and 0.214 ± 0.01, respectively). Samples S2 and S3 displayed a lower Browning index than the other carob syrups samples (Kmix 420 nm values of 0.146 ± 0.01 and 0.100 ± 0.01, respectively).

### 3.2. Identification of Individual Phenolic Compounds in Carob Syrups by High-Resolution Mass Spectrometry

The qualitative and quantitative profile of phenolic compounds in undigested carob syrups was determined by high-resolution mass spectrometry. The mass spectral data of identified phenolic compounds can be found in Supplementary Table S1, whereas the quantitative data are shown in Table 1.

**Figure 2:**
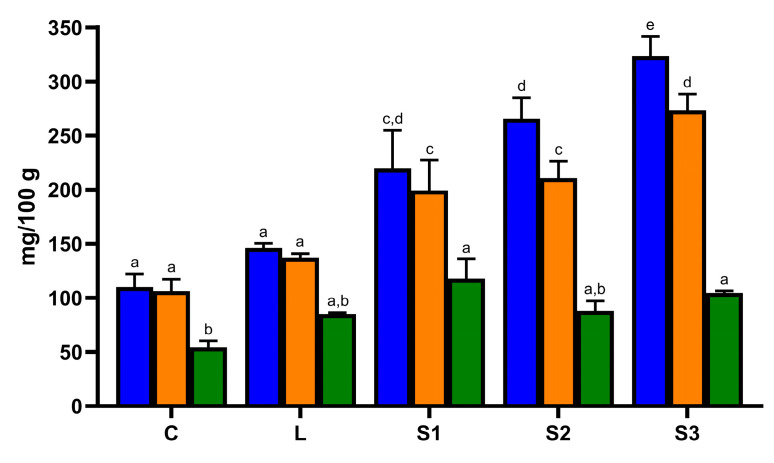
Total phenolic compounds (blue bars), total hydroxybenzoic acids (orange bars), and gallic acid (green bars) concentration in the different carob syrups determined by high-resolution mass spectrometry. The total phenolic compound concentration was calculated by summing the amount of each identified phenolic compound. The total hydroxybenzoic acid concentration was calculated by summing the amount of each identified hydroxybenzoic acid. Different letters mean significant differences (*p* < 0.05).

Considering all the samples, a total of 75 phenolic compounds belonging to different classes were identified in undigested carob syrups. The most representative class of phenolic compounds was represented by hydroxybenzoic acids (27 compounds), followed by hydroxycinnamic acids (13 compounds). Several flavonoids belonging to the classes of flavonols (11 compounds), flavanones (9 compounds), flavan-3-ols (8 compounds), and flavones (5 compounds) were also identified. Finally, two additional phenolic compounds, ellagic acid and one isomer of dihydroxyphenyl-acetic acid, were detected as well. Previous works identified 8 to 12 phenolic compounds in carob syrups [8,9,13]. Therefore, the present work represented the most extensive phenolic fingerprint of carob syrup until now. Great variability in the phenolic content among the different carob syrups was observed. The concentration of total phenolic compounds identified by mass spectrometry ranged between 110.13 ± 11.87 mg/100 g of carob syrup in sample C to 323.46 ± 18.77 mg/100 g of carob syrup in sample S3 (Figure 2).

The total amount of phenolic compounds was in the range of those reported in articles previously published, where phenolic compounds were quantified by liquid chromatography or mass spectrometry [8,9,13]. In all the samples, the quantitatively most important class of phenolic compounds was represented by hydroxybenzoic acids (Figure 2 and Table 1). In carob syrups C and L, hydroxybenzoic acids represented almost entirely the phenolic profiles, where they accounted for 96.52% and 93.86% of total phenolic compounds, respectively. In carob syrups S1 and S3, the incidence of hydroxybenzoic acids was lower and equal to 90.76% and 84.51% of total phenolic compounds, respectively. Nevertheless, in carob syrup S2, hydroxybenzoic acids incidence was 79.33%. In this sample, an appreciable amount of flavonols (7.25% of total phenolic compounds) and, especially, ellagic acid (10.37% of total phenolic compounds) were also found (Table 1). According to previously published data, in carob syrups C, L, and S1, gallic acid was the predominant compound, accounting for 50% or more of all phenolic compounds (Figure 2 and Table 1) [8,9,13]. In absolute terms, carob syrup S1 also contained the highest amount of gallic acid (117.73 ± 18.57 mg/100 g of syrup), whereas sample C showed the lowest (54.28 ± 5.97 mg/100 g of syrup). These samples were also relatively rich in isomers of gallic acid-O-hexoside and gallic acid-O-hexoside-O-hexoside (Table 1). In samples S2 and S3, gallic acid was still quantitatively the most important phenolic compound but represented 33.09% and 32.40% of total phenolic compounds, respectively. Those samples also provided the highest amount of ellagic acid and were also rich in some isomers of gallic acid-O-hexoside and gallic acid-O-hexoside-O-hexoside (Table 1). The most abundant flavonol was quercetin-3-O-rhamnoside, especially in samples S2 and S3, where it reached concentrations of 12.62 ± 0.65 and 19.12 ± 1.16 mg/100 g of carob syrup (Table 1). The highest amount of hydroxycinnamic acids was found in carob syrups S1 and S2, whereas flavones and flavanones were found only in trace amounts in all the samples and flavan-3-ols only in samples S1, S2, and S3 but always at very low concentration (Table 1).

### 3.3. Correlation Analysis among the Variables in Carob Syrups

Pearson analysis was carried out to look for relationships between the variables, i.e., total phenolic compounds, anti-oxidant activity assays, Browning index, and mass spectrometry data. The Pearson coefficient r and the respective *p*-values can be found in Supplementary Tables S3 and S4.

As observed, the Browning index was negatively correlated with the total concentration of phenolic compounds determined by mass spectrometry and the concentration of gallic acid. Moreover, the Browning index was also negatively correlated with the concentration of the gallic acid derivatives (such as gallic acid-mono-hexosides and gallic acid-di-hexosides) and the majority of glycosylated flavonoids. Since the Browning index is a measure of the extent of the thermal treatment, these results suggested that the more intense the heat treatment to which the syrup is subjected, the greater the degradation of the phenolic compounds. Previous studies suggested that gallic acid underwent degradation induced by thermal treatment [29,33]. Furthermore, thermal treatments induced flavonoid deglycosylation, as previously observed for flavonols [34,35]. Moreover, phenolic compounds could be incorporated into the melanoidin structure during Maillard reaction progression [36]. No correlation was found between the Browning index and the Folin–Ciocalteau data, whereas these last data were positively correlated with the total phenolic determined by mass spectrometry. Therefore, Folin–Ciocalteau reactivity seemed to be more dependent on the phenolic compounds content than on the brown melanoidins, although even the latter can react in a dose-dependent manner with Folin’s reagent [37]. As expected, a strong positive relationship was found between the Folin–Ciocalteau data and the anti-oxidant activity assay data. The Browning index did not correlate with data from the FRAP assay, whereas negative correlations were found with the ABTS data and the ability of the samples to hinder Fenton’s reaction. Moreover, data from anti-oxidant activity assays were positively correlated with total phenolic compounds determined by mass spectrometry and with the concentration of the majority of individual phenolic compounds, suggesting that phenolic compounds were the major determinant of the anti-oxidant properties of carob syrups, especially for ABTS radical scavenging ability and the capability to prevent Fenton reaction.

### 3.4. Effect of In Vitro Gastro–Intestinal Digestion on Total Phenolic Compounds and Anti-Oxidant Properties of Carob Syrups

The effect of in vitro gastro–intestinal digestion on total phenolic compounds and anti-oxidant activity assays on carob syrups is depicted in Figure 1. As reported in Figure 1A, the trend of total phenolic content determined by Folin–Ciocalteau assays was different depending on the carob syrup samples. For samples C and L, in vitro digestion resulted in a small and non-significant (*p* > 0.05) decrease in the total phenolic content value, whereas in samples S1, S2, and S3, the total phenolic content significantly increased after in vitro digestion. A similar trend was also observed for ABTS radical scavenging capacity, whereas the ferric-reducing ability decreased significantly (*p* < 0.05) in all the carob syrup samples following in vitro gastro–intestinal digestion (Figure 1B,C). Finally, a significant (*p* < 0.05) decrease in the capacity to inhibit the Fenton reaction was recorded after in vitro digestion of carob syrups L and S3, whereas no significant differences were noted between the undigested and digested samples of carob syrups C, S1, and S2 (Figure 1D). No studies are present in the literature about the effect of in vitro gastro–intestinal digestion on carob syrups. In one study, the in vitro digestion of carob pod resulted in a decrease in total phenolic content and radical scavenging activity [12]. In another study on carob flour, the behavior of total phenolic content and ABTS radical scavenging activity during in vitro digestion was strongly dependent on the particle size of carob flour [38]. 

### 3.5. Bioaccessibility of Individual Phenolic Compounds in Carob Syrups after In Vitro Gastro–Intestinal Digestion

In vitro gastro–intestinal digestion of carob syrups resulted in a general decrease in phenolic compounds concentration in all the analyzed samples (Table 1 and Table 2).

**Figure 3:**
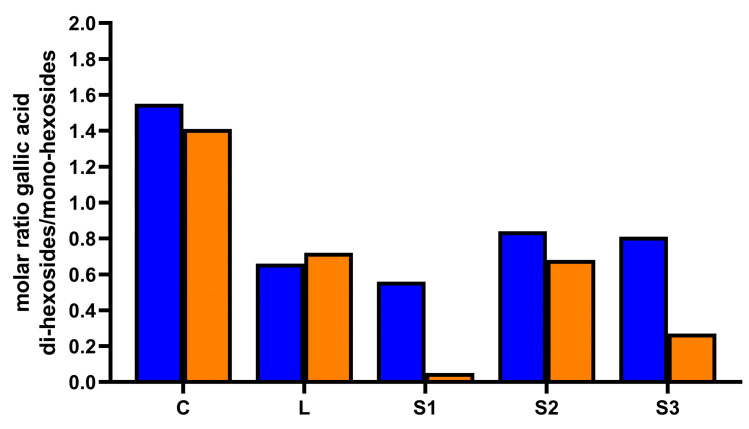
Molar ratio between gallic acid di-hexosides and gallic acid mono-hexosides calculated before (blue bars) and after (orange bars) in vitro gastro–intestinal digestion. The ratio was calculated by summing the amount of each individual gallic acid di-hexoside isomer divided by the sum of each individual gallic acid mono-hexoside isomer.

The highest amount of phenolic compounds detected after in vitro digestion was found for carob syrup S3 (301.61 ± 5.42 mg/100 g of carob syrup), followed by carob syrup S2 (185.95 ± 2.82 mg/100 g of carob syrup) and carob syrup S1 (118.24 ± 1.57 mg/100 g of carob syrup). The carob syrups C and L displayed a similar amount of bio-accessible phenolic compounds (75.55 ± 2.18 mg/100 g of carob syrup and 81.70 ± 1.24 mg/100 g of carob syrup, respectively) at the end of the gastro–intestinal digestion. The bioaccessibility index of total phenolic compounds was the highest for sample S3 (93.25%), whereas in the other carob syrup samples was lower and quite similar, ranging between 53.83% and 69.98% in carob syrups S1 and S2, respectively. The behavior of hydroxybenzoic acids during in vitro gastro–intestinal digestion was different depending on the considered sample (Table 2). The lowest bioaccessibility of total hydroxybenzoic acids was found for carob syrup S3 (17.56%), whereas the highest was for carob syrup C (54.62%). As reported above, among hydroxybenzoic acids, gallic acid was the most concentrated compound in all the undigested carob syrups (Table 1). However, a strong degradation of gallic acid was observed in all the samples after in vitro digestion (Table 2). The lowest bioaccessibility indexes (which means the highest degradation) were found for samples S1 (0.52%) and S3 (0.94%), which contained the highest amounts of gallic acid before digestion (Table 1 and Table 2). Carob syrup C displayed the lowest gallic acid concentration before digestion and the lowest degradation rate, with a bioaccessibility index of 35.59% (Table 1 and Table 2). Carob syrups L and S2, which exhibited a similar amount of gallic acid before digestion, featured a similar gallic acid degradation rate after in vitro digestion with a bioaccessibility index of 21.44% and 22.40%, respectively (Table 1 and Table 2). Therefore, the extent of gallic acid degradation seemed related to the initial concentration before digestion: the higher the initial concentration, the greater the degradation. Furthermore, also the different isomers of gallic acid-O-hexoside and gallic acid-O-hexoside-O-hexoside underwent degradation during in vitro digestion to a different extent depending on the sample. Once again, the highest degradation rate was found for carob syrups S1 and S3 (Table 1 and Table 2). The degradation of gallic acid mono- and di-hexosides may be a consequence of their deglycosylation occurring during in vitro digestion. Previous studies have suggested that gallic acid-hexosides were unstable under acidic conditions and underwent deglycosylation reaction, releasing gallic acid during in vitro gastric digestion [39,40,41]. The decrease in the molar ratio between gallic acid di-hexosides and gallic acid mono-hexosides suggested that gallic acid di-hexosides were hydrolyzed in the corresponding mono-hexosides during in vitro digestion. This decrease was particularly evident in carob syrups S1 and S3, where the extent of gallic acid and derivatives degradation was more extreme (Figure 3). 

**Figure 4:**
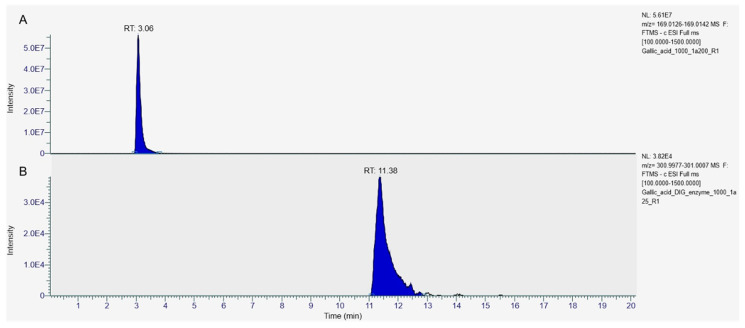
Extracted ion chromatograms (EICs) of gallic and ellagic acids before and after in vitro digestion of a solution of pure gallic acid. (**A**) EIC of gallic acid extracted at *m/z* 169.0392 (tolerance ± 5 ppm) from a gallic acid solution before in vitro gastro–intestinal digestion. (**B**) EIC of ellagic acid extracted at *m/z* 300.9992 (tolerance ± 5 ppm) from a gallic acid solution after in vitro gastro–intestinal digestion. The EICs shown are representative of three independent experiments.

Similarly, gallic acid mono-hexosides can be deglycosylated to gallic acid during the gastric phase of digestion, as already suggested [39,40,41]. However, gallic acid degradation occurring during the intestinal phase of the digestion hid the expected increase in its concentration due to the hydrolysis of the glycosylated derivatives. Previously, it had been demonstrated that gallic acid is stable during gastric digestion but degraded under intestinal conditions [42]. Similarly, gallic acid was found stable during gastric digestion of carob liqueur obtained by aqueous infusion but was almost completely degraded during the intestinal phase of the digestion [43]. Gallic acid degradation during in vitro digestion was strictly related to an increase in the concentration of ellagic acid (Table 1 and Table 2). The increase in ellagic acid concentration was the highest in samples S1 and S3 (an increase of 15.61 and 19.20 times, respectively), where the gallic acid degradation was more severe. Previous studies suggested that gallic acid has undergone oxidative polymerization in an alkaline media with ellagic acid as an intermediate [44,45]. The oxidative polymerization of gallic acid proceeds via the formation of a C–C dimer of gallic acid, followed by the elimination of two water molecules to give ellagic acid [44,45,46]. The formation of ellagic acid from gallic acid was confirmed by digesting a solution of the gallic acid standard at the same concentration as found in the carob syrup sample S3 by applying the same INFOGEST protocol (Figure 4).

**Figure 5:**
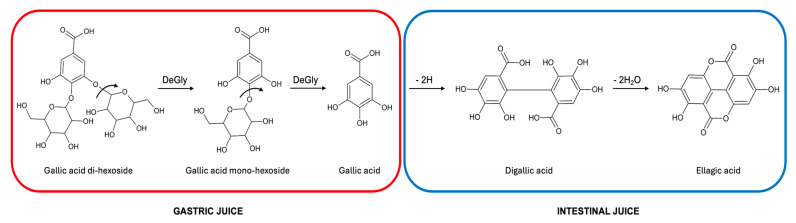
Gallic acid and gallic acid-hexosides degradation pathway during in vitro gastro–intestinal digestion. DeGly—deglycosylation.

At the end of the in vitro digestion, 98.2% of gallic acid was degraded (versus 99% of degradation in the S3 sample), and the solution turned into a blue/green color, which indicated the formation of colored polymers. The amount of ellagic acid formed was much less than that observed after digestion of carob syrup S3 (about 8.2 μmol/L in digested gallic acid with respect to 7853 μmol/L in digested sample S3). Overall, these results confirmed the pathway of ellagic acid formation from gallic acid degradation and suggested that the food matrix (i.e., the carob syrup) may protect ellagic acid from further oxidative polymerization reactions. Figure 5 displays the proposed pathway of gallic acid and gallic acid-derivative degradation during in vitro gastro–intestinal digestion based on previously reported data and the data presented here.

**Figure 6:**
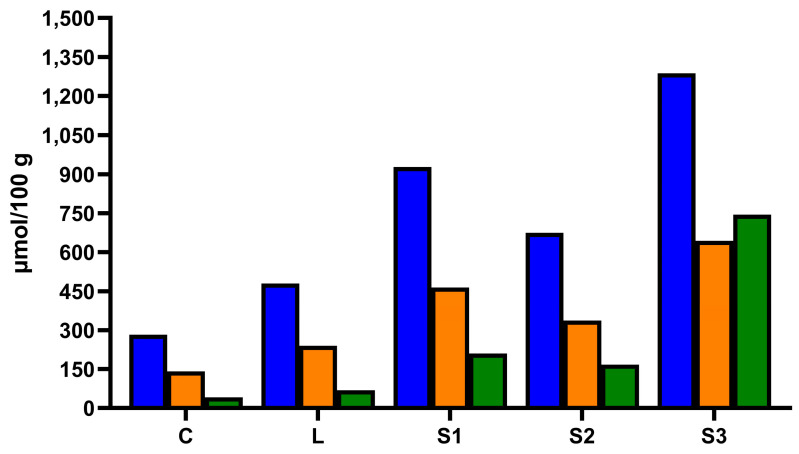
Molar conversion of gallic acid to ellagic acid during in vitro gastro–intestinal digestion. Blue bars show the sum of the concentrations of gallic acid, gallic acid mono-hexosides, and gallic acid di-hexosides in the different carob syrups before in vitro digestion. Orange bars show the expected ellagic acid concentration after in vitro gastro–intestinal digestion if all gallic acid derivatives were converted to ellagic acid (molar ratio gallic acid:ellagic acid of 2:1). Green bars show the ellagic concentration in the different carob syrups after in vitro digestion.

On the basis of the postulated degradation pathway, two moles of gallic acid are needed to yield one mol of ellagic acid. The mass balance (data are expressed as μmol/100 g) between gallic acid degradation (sum of lost gallic acid, gallic acid mono-hexosides, and gallic acid di-hexosides) and ellagic acid production, together with the expected concentration of ellagic acid calculated on the basis of gallic acid degradation is reported in Figure 6.

As shown, with the exception of carob syrup S3, the amount of produced ellagic acid was always lower than that expected on the basis of gallic acid degradation, ranging between 28.46% and 49.77% than expected in samples L and S1, respectively. Since the intermediate C–C dimer of gallic acid was not found in these samples, it is probable that the formed ellagic acid was further subjected to oxidative polymerization, generating new unknown compounds. In carob syrup S3, the amount of detected ellagic acid was slightly higher than expected, suggesting that during the digestion of carob syrup S3, ellagic acid was protected by the food matrix to further oxidative reaction. It is important to note that this sample exhibited the highest anti-oxidant activity in all the assays (Figure 1). The slightly higher than expected value of ellagic acid concentration in sample S3 may be due to the oxidative degradation of other gallic acid derivatives detected (such as gallic acid-glucuronide isomers and malonyl-gallic acid) or not detected by mass spectrometry. Regarding the bioaccessibility of hydroxycinnamic acids, the values were higher than 100% for all the digested carob samples (Table 2). Some hydroxycinnamic acids, such as coumaric acid and its isomers, and some isomers of ferulic acid-O-hexoside were found in higher concentrations in digested samples with respect to the undigested carob syrups. In addition, some hydroxybenzoic acids such as hydroxybenzoic acid and its isomers and the different isomers of di-hydroxybenzoic acid, as well as their glycosylated forms, were found in higher amounts after in vitro digestion with respect to undigested samples. It is well known that these compounds may be incorporated into the melanoidin structure during the Maillard reaction and can be released following in vitro gastro–intestinal digestion [47]. The bioaccessibility index of flavan-3-ols and flavonols was quite low and always less than 15% and 45%, respectively. These results are in agreement with previous data [35,48,49].

### 3.6. Correlation Analysis among the Variables in Digested Carob Syrups

After in vitro digestion, a strong correlation was still found between the Folin–Ciocalteau data and the different anti-oxidant activity assays (Supplementary Tables S5 and S6). Once again, the amount of total phenolic compounds determined by mass spectrometry was positively correlated with the Folin–Ciocalteau data and the different anti-oxidant activity assays (with the exception of the inhibition of the Fenton reaction), suggesting that also in the in vitro digested samples phenolic compounds were the major contributor to the anti-oxidant activity of carob syrup. Anti-oxidant activity and Folin–Ciocalteau data were also positively correlated with the major phenolic compounds present in the digested samples, including ellagic acid. This can explain the higher Folin–Ciocalteau values found in digested samples S1, S2, and S3 with respect to the undigested samples despite the decrease in the amount of phenolic compounds detected by mass spectrometry. In fact, in carob syrups, the major phenolic compound was gallic acid, whereas ellagic acid was quantitatively the predominant compound in in vitro digested carob syrups (Table 1 and Table 2). Ellagic acid showed a greater reactivity (about 16% more than gallic acid when tested at the same concentration) with the Folin–Ciocalteau reagent with respect to gallic acid.

## 4. Conclusions

Mass spectrometry analysis revealed that carob syrups were particularly rich in hydroxybenzoic acids and especially gallic acid. The amount of these compounds was different among the five tested samples and was negatively related to the Browning index, which is a measure of the extent of the thermal treatment. Therefore, the thermal treatment to which the carob is subjected for the preparation of the syrup is of paramount importance in determining the phenolic profiles of the syrup itself. The phenolic compound profiles and the anti-oxidant activity of carob syrups were significantly affected by the in vitro gastro–intestinal digestion. In particular, in vitro digestion caused a partial degradation of the major phenolic compounds found in carob syrups. Gallic acid was mostly affected by the digestion procedure and underwent oxidative polymerization, producing ellagic acid, which was the predominant compound in in vitro digested carob syrups. Therefore, the results reported in this study suggest that ellagic acid, rather than gallic acid, may be responsible for the effects of carob syrups on the gastro–intestinal tract. Since ellagic acid is thoroughly metabolized by gut microbiota in urolithins, a class of phenolic metabolites with pleiotropic health effects, carob syrup could be considered an unconventional source of these phenolic metabolites. Whether these transformations also occur in vivo remains to be demonstrated since there are no studies about the in vivo phenolic compound metabolism after ingestion of carob syrups.

## Figures and Tables

**Table 1:** Quantitative data of phenolic compounds identified in the different carob syrups by high-resolution mass spectrometry. Data are expressed as mg/100 g of carob syrup.

Carob Syrup					
Compound	C	L	S1	S2	S3
**Hydroxybenzoic acids**					
Hydroxybenzoic acid isomer 1	0.07 ± 0.02	0.13 ± 0.01	0.30 ± 0.18	0.25 ± 0.07	0.10 ± 0.01
Hydroxybenzoic acid isomer 2	0.30 ± 0.02	0.47 ± 0.03	0.48 ± 0.09	0.43 ± 0.05	0.35 ± 0.01
Hydroxybenzoic acid isomer3	0.33 ± 0.01	0.19 ± 0.00	0.12 ± 0.02	0.09 ± 0.01	0.14 ± 0.00
Dihydroxybenzoic acid isomer	n.d.	0.31 ± 0.02	0.65 ± 0.27	0.58 ± 0.17	0.24 ± 0.10
Protocatechuic acid	0.15 ± 0.01	0.20 ± 0.01	0.34 ± 0.07	0.29 ± 0.05	0.22 ± 0.06
Gentisic acid	0.21 ± 0.01	0.51 ± 0.01	0.17 ± 0.03	0.09 ± 0.04	0.14 ± 0.02
Gallic acid	54.28 ± 5.97	85.03 ± 1.24	117.73 ± 18.57	87.93 ± 9.19	104.79 ± 1.73
Malonyl-gallic acid	0.16 ± 0.02	0.26 ± 0.01	0.41 ± 0.33	0.55 ± 0.31	1.38 ± 0.52
Hydroxybenzoic acid-O-hexoside isomer 1	n.d.	n.d.	0.19 ± 0.03	0.17 ± 0.02	0.16 ± 0.01
Hydroxybenzoic acid-O-hexoside isomer 2	n.d.	0.05 ± 0.00	0.14 ± 0.01	0.10 ± 0.03	0.10 ± 0.03
Dihydroxybenzoic acid-O-hexoside isomer	0.27 ± 0.03	0.27 ± 0.02	n.d.	n.d.	n.d.
Hydroxy-methoxybenzoic acid-O-hexoside isomer	5.02 ± 0.50	8.66 ± 0.35	16.56 ± 2.20	9.61 ± 0.25	5.88 ± 0.14
Gallic acid-O-hexoside isomer 1	2.29 ± 0.08	2.21 ± 0.09	3.32 ± 0.51	8.53 ± 0.05	12.21 ± 2.08
Gallic acid-O-hexoside isomer 2	1.90 ± 0.21	1.97 ± 0.07	2.91 ± 0.30	5.30 ± 1.06	9.74 ± 1.01
Gallic acid-O-hexoside 3 isomer	4.53 ± 0.25	6.02 ± 0.16	9.84 ± 0.21	13.74 ± 0.52	12.55 ± 0.46
Gallic acid-O-hexoside isomer 4	4.56 ± 0.63	6.27 ± 0.14	9.95 ± 0.05	15.51 ± 0.14	10.40 ± 1.07
Gallic acid-O-hexoside isomer 5	0.71 ± 0.08	0.39 ± 0.00	2.06 ± 0.64	2.59 ± 0.11	25.78 ± 1.94
Gallic acid-O-glucuronide isomer 1	0.67 ± 0.08	0.87 ± 0.12	n.d.	1.45 ± 0.14	1.64 ± 0.06
Gallic acid-O-glucuronide isomer 2	0.42 ± 0.08	0.45 ± 0.01	0.65 ± 0.23	0.77 ± 0.07	0.86 ± 0.05
Gallic acid-O-glucuronide isomer 3	0.50 ± 0.14	0.58 ± 0.03	0.80 ± 0.27	0.66 ± 0.58	0.95 ± 0.14
Gallic acid-O-glucuronide isomer 4	n.d.	0.5 ± 0.02	0.74 ± 0.02	0.72 ± 0.03	0.77 ± 0.03
Syringic acid-O-hexoside isomer	1.43 ± 0.19	2.06 ± 0.13	3.23 ± 0.55	2.93 ± 0.04	2.84 ± 0.03
Vanillic acid-O-hexoside-pentoside isomer 1	6.87 ± 0.20	7.45 ± 0.39	13.11 ± 2.22	20.09 ± 0.41	25.08 ± 1.50
Vanillic acid-O-hexoside-pentoside isomer 2	n.d.	1.30 ± 0.05	n.d.	n.d.	n.d.
Gallic acid-O-hexoside-O-hexoside isomer 1	12.77 ± 1.38	7.47 ± 0.29	8.98 ± 0.71	22.05 ± 1.66	31.38 ± 1.57
Gallic acid-O-hexoside-O-hexoside isomer 2	8.58 ± 0.96	3.45 ± 0.23	6.10 ± 0.27	15.49 ± 0.43	24.17 ± 1.60
Gallic acid-O-hexoside-O-hexoside-O-pentoside	0.29 ± 0.11	0.21 ± 0.01	0.61 ± 0.15	0.87 ± 0.09	1.50 ± 0.62
**Total hydroxybenzoic acids**	**106.30 ± 10.99**	**137.37 ± 3.44**	**199.37 ± 27.94**	**210.79 ± 15.53**	**497.76 ± 28.35**
**Hydroxycinnamic acids**					
Hydroxycinnamic acid isomer 1	n.d.	n.d.	0.17 ± 0.07	0.18 ± 0.06	0.10 ± 0.02
Hydroxycinnamic acid isomer 2	n.d.	n.d.	0.16 ± 0.08	0.14 ± 0.07	0.06 ± 0.02
p-Coumaric acid	0.27 ± 0.01	0.83 ± 0.04	1.03 ± 0.41	1.07 ± 0.41	0.86 ± 0.53
Ferulic acid	n.d.	0.14 ± 0.01	0.13 ± 0.10	0.29 ± 0.15	0.17 ± 0.07
Caffeoyl-hexose isomer 1	n.d.	0.06 ± 0.00	0.07 ± 0.01	0.09 ± 0.01	0.08 ± 0.02
Caffeoyl-hexose isomer 2	n.d.	0.14 ± 0.01	0.05 ± 0.01	0.02 ± 0.00	0.02 ± 0.01
Caffeoyl-hexose isomer 3	n.d.	0.18 ± 0.00	0.05 ± 0.01	0.03 ± 0.01	0.03 ± 0.00
Ferulic acid-O-hexoside isomer 1	n.d.	0.22 ± 0.01	0.39 ± 0.06	0.43 ± 0.05	0.29 ± 0.08
Ferulic acid-O-hexoside isomer 2	n.d.	0.18 ± 0.02	0.24 ± 0.07	0.14± 0.01	0.11 ± 0.04
Ferulic acid-O-hexoside isomer 3	n.d.	0.60 ± 0.04	0.23 ± 0.04	0.18± 0.02	0.18 ± 0.01
Ferulic acid-O-hexoside isomer 4	n.d.	0.52 ± 0.06	0.20 ± 0.03	0.16 ± 0.02	0.16 ± 0.01
Dimethoxy-hydroxycinnamic acid-O-hexoside isomer	n.d.	0.24 ± 0.05	0.68 ± 0.13	0.93 ± 0.12	0.78 ± 0.04
Coumaric acid-O-hexoside-pentoside	n.d.	n.d.	0.60 ± 0.10	n.d.	n.d.
**Total hydroxycinnamic acids**	**0.27 ± 0.01**	**3.11 ± 0.24**	**4.03 ± 1.11**	**11.87 ± 1.75**	**2.83 ± 0.84**
**Flavanols**					
Epicatechin	n.d.	n.d.	n.d.	0.04 ± 0.00	0.44 ± 0.04
Catechin	n.d.	n.d.	0.04 ± 0.06	0.01 ± 0.00	0.09 ± 0.02
Epigallocatechin	n.d.	n.d.	0.25 ± 0.44	0.04 ± 0.00	0.66 ± 0.09
Gallocatechin	n.d.	n.d.	0.10 ± 0.11	0.03 ± 0.03	0.18 ± 0.05
Epicatechin-3-O-gallate	n.d.	n.d.	0.02 ± 0.00	0.02 ± 0.00	0.18 ± 0.02
Epigallocatechin-3-O-gallate	n.d.	0.00 ± 0.01	0.04 ± 0.06	0.02 ± 0.00	0.11 ± 0.01
Epigallocatechin gallate isomer	n.d.	n.d.	n.d.	0.02 ± 0.00	0.23 ± 0.01
Procyanidin-type B dimer isomer	n.d.	n.d.	0.16 ± 0.27	n.d.	0.50 ± 0.02
**Total flavanols**	**n.d.**	**0.01 ± 0.01**	**0.61 ± 0.94**	**0.19 ± 0.05**	**2.38 ± 0.25**
**Flavanones**					
Naringenin isomer	n.d.	0.01 ± 0.00	0.02 ± 0.01	0.02 ± 0.00	0.04 ± 0.01
Naringenin	n.d.	n.d.	0.03 ± 0.04	0.04 ± 0.1	0.09 ± 0.2
Tetra-hydroxyflavanone isomer	n.d.	n.d.	0.02 ± 0.03	0.02 ± 0.00	0.07 ± 0.01
Naringenin-O-hexoside isomer 1	n.d.	n.d.	0.08 ± 0.07	0.06 ± 0.02	0.16 ± 0.01
Naringenin-O-hexoside isomer 2	n.d.	n.d.	0.11 ± 0.10	0.08 ± 0.01	0.19 ± 0.02
Naringenin-O-hexoside isomer 3	0.02 ± 0.00	0.01 ± 0.00	0.32 ± 0.26	0.27 ± 0.02	0.69 ± 0.08
Tetra-hydroxyflavanone-O-hexoside isomer 1	n.d.	0.06 ± 0.01	0.17 ± 0.05	0.30 ± 0.01	0.58 ± 0.04
Tetra-hydroxyflavanone-O-hexoside isomer 2	n.d.	n.d.	0.09 ± 0.05	0.06 ± 0.01	0.19 ± 0.02
Tetra-hydroxyflavanone-O-hexoside isomer 3	n.d.	0.01 ± 0.00	0.06 ± 0.08	0.04 ± 0.01	0.19 ± 0.02
**Total flavanones**	**0.02 ± 0.00**	**0.09 ± 0.01**	**0.90 ± 0.68**	**0.89 ± 0.07**	**2.20 ± 0.23**
**Flavones**					
Luteolin	n.d.	n.d.	0.12 ± 0.17	0.18 ± 0.02	0.35 ± 0.03
Tri-hydroxy-methoxyflavone isomer	n.d.	n.d.	0.01 ± 0.00	0.11 ± 0.01	0.09 ± 0.01
Apigenin-7-O-glucoside	0.01 ± 0.00	0.02 ± 0.00	0.26 ± 0.29	0.22 ± 0.2	0.66 ± 0.7
Luteolin-O-rhamnoside	n.d.	0.01 ± 0.00	0.38 ± 0.30	0.44 ± 0.05	0.57 ± 0.42
Luteolin-7-O-glucoside	0.01 ± 0.00	0.02 ± 0.00	0.25 ± 0.27	0.25 ± 0.00	0.58 ± 0.02
**Total flavones**	**0.02 ± 0.00**	**0.06 ± 0.00**	**1.03 ± 1.03**	**5.51 ± 0.58**	**7.69 ± 0.75**
**Flavonols**					
Quercetin	n.d.	n.d.	0.19 ± 0.13	0.25 ± 0.01	0.37 ± 0.03
Methyl-quercetin isomer	n.d.	n.d.	0.03 ± 0.01	0.13 ± 0.02	0.06 ± 0.01
Isorhamnetin	n.d.	n.d.	0.01 ± 0.01	0.02 ± 0.00	0.02 ± 0.00
Myricetin	n.d.	n.d.	0.06 ± 0.06	0.10 ± 0.00	0.13 ± 0.01
Quercetin-3-O-pentoside	n.d.	n.d.	0.17 ± 0.17	0.30 ± 0.03	0.40 ± 0.03
Quercetin-3-O-pentoside isomer 1	n.d.	n.d.	0.24 ± 0.29	0.40 ± 0.08	0.59 ± 0.03
Quercetin-3-O-pentoside isomer 2	n.d.	n.d.	0.22 ± 0.25	0.43 ± 0.04	0.64 ± 0.12
Quercetin-3-O-rhamnoside	0.01 ± 0.00	0.06 ± 0.05	4.14 ± 0.39	12.62 ± 0.65	19.12 ± 1.16
Myricetin-O-rhamnoside	0.03 ± 0.04	0.10 ± 0.02	0.95 ± 0.08	3.99 ± 0.33	3.18 ± 0.21
Quercetin-3-O-glucoside	0.02 ± 0.00	n.d.	0.79 ± 0.82	0.99 ± 0.08	1.96 ± 0.21
Quercetin glucoside isomer	n.d.	n.d.	0.06 ± 0.06	0.04 ± 0.03	0.13 ± 0.01
**Total flavonols**	**0.06 ± 0.04**	**0.17 ± 0.06**	**6.87 ± 2.29**	**19.27 ± 1.28**	**26.62 ± 1.82**
**Others**					
Dihydroxyphenylacetic acid isomer	n.d.	2.76 ± 0.36	2.54 ± 0.88	2.19 ± 0.99	1.52 ± 0.19
Ellagic acid	3.46 ± 0.81	2.64 ± 0.10	4.33 ± 0.24	27.56 ± 0.35	12.31 ± 0.03
**Total others**	**3.46 ± 0.81**	**5.40 ± 0.47**	**6.87 ± 1.12**	**29.75 ± 1.34**	**13.83 ± 0.22**
**Total phenolic by MS**	**110.13 ± 11.87**	**146.21 ± 4.24**	**219.68 ± 35.11**	**265.73 ± 19.31**	**323.46 ± 18.77**

n.d. means compound not detected in the sample.

**Table 2:** Quantitative data of phenolic compounds identified in the different carob syrups by high-resolution mass spectrometry after in vitro gastro–intestinal digestion. Data are expressed as mg/100 g of carob syrup. BI means bioaccessibility index.

Compound	Carob Syrup									
	C		L		S1		S2		S3	
	After Digestion	BI (%)	After Digestion	BI (%)	After Digestion	BI (%)	After Digestion	BI (%)	After Digestion	BI (%)
**Hydroxybenzoic acids**										
Hydroxybenzoic acid isomer 1	0.09 ± 0.00	118.1	0.12 ± 0.00	86.3	0.14 ± 0.01	48.1	0.06 ± 0.01	24.6	0.07 ± 0.00	75.1
Hydroxybenzoic acid isomer 2	0.31 ± 0.02	105.3	0.43 ± 0.01	91.1	0.57 ± 0.01	118.7	0.50 ± 0.01	115.4	0.49 ± 0.00	142.0
Hydroxybenzoic acid isomer 3	0.44 ± 0.01	131.1	0.28 ± 0.00	147.4	0.14 ± 0.00	121.0	0.12 ± 0.01	127.4	0.19 ± 0.01	135.3
Dihydroxybenzoic acid isomer	0.04 ± 0.00	n.f.	0.13 ± 0.01	40.7	0.09 ± 0.01	13.3	0.07 ± 0.01	12.3	0.07 ± 0.01	29.9
Protocatechuic acid	0.16 ± 0.01	106.9	0.20 ± 0.00	97.9	1.40 ± 0.09	416.1	0.45 ± 0.00	157.3	0.68 ± 0.02	307.1
Gentisic acid	0.22 ± 0.01	105.0	0.47 ± 0.01	92.7	0.17 ± 0.01	98.9	0.10 ± 0.00	108.4	0.15 ± 0.02	112.3
Gallic acid	19.32 ± 0.62	35.6	18.23 ± 0.76	21.4	0.61 ± 0.03	0.5	19.70 ± 0.54	22.4	0.98 ± 0.03	0.9
Malonyl-gallic acid	0.15 ± 0.00	95.7	0.28 ± 0.12	106.9	0.08 ± 0.00	20.6	0.23 ± 0.01	42.6	0.12 ± 0.00	8.5
Hydroxybenzoic acid-O-hexoside isomer 1	n.d.	n.d.	n.d.	n.d.	0.28 ± 0.01	145.6	0.24 ± 0.00	146.1	0.30 ± 0.01	191.6
Hydroxybenzoic acid-O-hexoside isomer 2	n.d.	n.d.	0.14 ± 0.02	275.3	0.16 ± 0.01	116.2	0.14 ± 0.01	134.7	0.15 ± 0.00	153.7
Dihydroxybenzoic acid-O-hexoside isomer	0.40 ± 0.01	147.9	0.28 ± 0.01	102.2	n.d.	n.d.	n.d.	n.d.	0.44 ± 0.02	n.f.
Hydroxy-methoxybenzoic acid-O-hexoside isomer	4.90 ± 0.08	97.6	7.40 ± 0.46	85.4	16.19 ± 0.78	97.8	8.22 ± 0.20	85.5	6.68 ± 0.07	113.7
Gallic acid-O-hexoside isomer 1	1.59 ± 0.03	69.2	1.07 ± 0.02	48.6	0.05 ± 0.01	1.5	4.47 ± 0.03	52.4	1.28 ± 0.06	10.5
Gallic acid-O-hexoside isomer 2	1.13 ± 0.03	59.3	0.82 ±0.08	41.6	0.04 ± 0.00	1.2	3.35 ± 0.12	63.2	0.87 ± 0.08	8.9
Gallic acid-O-hexoside isomer 3	3.40 ± 0.01	75.0	3.00 ±0.06	49.9	1.48 ± 0.07	15.1	6.42 ± 0.11	46.7	4.93 ± 0.03	39.2
Gallic acid-O-hexoside isomer 4	2.70 ± 0.09	59.3	2.52 ±0.11	40.2	0.07 ± 0.01	0.7	5.93 ± 0.25	38.2	0.60 ± 0.03	5.8
Gallic acid-O-hexoside isomer 5	0.57 ± 0.01	80.0	0.31 ±0.03	78.9	1.33 ± 0.06	64.4	2.22 ± 0.06	85.7	2.35 ± 0.11	9.1
Gallic acid-O-glucuronide isomer 1	0.45 ± 0.02	67.2	0.48 ± 0.02	54.7	0.02 ± 0.00	n.f.	0.71 ± 0.02	49.0	0.11 ± 0.01	6.8
Gallic acid-O-glucuronide isomer 2	0.26 ± 0.02	62.7	0.23 ± 0.01	50.8	0.02 ± 0.00	3.6	0.34 ± 0.02	44.8	0.06 ± 0.01	7.2
Gallic acid-O-glucuronide isomer 3	0.34 ± 0.02	67.4	0.28 ± 0.02	47.5	0.02 ± 0.00	2.0	0.36 ± 0.01	54.5	0.08 ± 0.00	8.4
Gallic acid-O-glucuronide isomer 4	0.23 ± 0.01	n.f.	0.25 ± 0.01	42.4	0.02 ± 0.00	2.9	0.23 ± 0.01	31.9	0.06 ± 0.00	7.8
Syringic acid-O-hexoside isomer	1.65 ± 0.03	115.7	1.99 ± 0.09	96.6	3.20 ± 0.26	99.1	2.58 ± 0.03	88.1	2.88 ± 0.10	101.5
Vanillic acid-O-hexoside-pentoside isomer 1	5.65 ± 0.17	82.2	6.31 ± 0.31	84.7	11.17 ± 0.81	85.2	15.25 ± 0.16	75.9	18.50 ± 0.45	73.78
Vanillic acid-O-hexoside-pentoside isomer 2	0.89 ± 0.03	n.f.	1.05 ± 0.08	80.4	0.91 ± 0.01	n.f.	2.34 ± 0.08	n.f.	3.18 ± 0.09	n.f.
Gallic acid-O-hexoside-O-hexoside isomer 1	8.11 ± 0.56	63.5	3.52 ± 0.10	47.1	0.06 ± 0.00	0.7	8.73 ± 0.02	39.6	1.16 ± 0.11	3.7
Gallic acid-O-hexoside-O-hexoside isomer 2	4.86 ± 0.27	56.7	1.88 ± 0.02	54.6	0.08 ± 0.00	1.3	6.17 ± 0.02	39.8	1.48 ± 0.08	6.1
Gallic acid-O-hexoside-O-hexoside-O-pentoside	0.21 ± 0.01	72.7	0.12 ± 0.01	59.4	n.d.	0.8	0.37 ± 0.00	42.1	0.12 ± 0.00	7.8
**Total hydroxybenzoic acids**	**58.06 ± 2.05**	**54.6**	**51.78 ± 2.38**	**37.7**	**38.30 ± 2.18**	**19.2**	**89.30 ± 1.74**	**42.4**	**47.99 ± 1.35**	**17.6**
**Hydroxycinnamic acids**										
Hydroxycinnamic acid isomer 1	0.05 ± 0.00	n.f.	0.06 ± 0.00	n.f.	0.26 ± 0.02	154.1	0.20 ± 0.00	109.1	0.10 ± 0.00	106.6
Hydroxycinnamic acid isomer 2	0.04 ± 0.00	n.f.	0.07 ± 0.00	n.f.	0.23 ± 0.00	137.6	0.11 ± 0.00	81.6	0.07 ± 0.00	116.2
p-Coumaric acid	0.56 ± 0.05	205.2	1.20 ± 0.05	143.2	1.75 ± 0.11	169.0	2.34 ± 0.04	218.8	1.27 ± 0.04	146.6
Ferulic acid	0.09 ± 0.00	n.f.	0.28 ± 0.12	200.3	0.08 ± 0.00	62.3	0.23 ± 0.01	82.2	0.12 ± 0.00	68.2
Caffeoyl-hexose isomer 1	n.d.	n.d.	0.05 ± 0.00	77.6	0.07 ± 0.00	90.5	0.08 ± 0.00	89.4	0.07 ± 0.00	87.9
Caffeoyl-hexose isomer 2	n.d.	n.d.	n.d.	n.d.	0.01 ± 0.00	18.1	n.d.	16.4	n.d.	7.3
Caffeoyl-hexose isomer 3	n.d.	n.d.	n.d.	n.d.	0.03 ± 0.00	51.5	0.02 ± 0.00	68.8	0.02 ± 0.00	80.5
Ferulic acid-O-hexoside isomer 1	n.d.	n.d.	0.27 ± 0.00	123.9	0.38 ± 0.04	97.7	0.46 ± 0.01	107.7	0.25 ± 0.01	88.7
Ferulic acid-O-hexoside isomer 2	n.d.	n.d.	0.19 ± 0.00	105.1	0.27 ± 0.01	110.5	0.17 ± 0.00	119.6	0.11 ± 0.00	99.0
Ferulic acid-O-hexoside isomer 3	n.d.	n.d.	0.16 ± 0.01	27.1	0.02 ± 0.00	9.4	0.07 ± 0.01	40.9	0.04 ± 0.00	22.8
Ferulic acid-O-hexoside isomer 4	n.d.	n.d.	0.16 ± 0.02	30.2	0.02 ± 0.00	8.6	0.06 ± 0.00	38.3	0.04 ± 0.00	23.1
Dimethoxy-hydroxycinnamic acid-O-hexoside isomer	n.d.	n.d.	2.26 ± 0.02	957.2	0.42 ± 0.01	62.2	0.89 ± 0.01	96.2	0.78 ± 0.00	99.6
Coumaric acid-O-hexoside-pentoside	0.19 ± 0.01	n.f.	0.16 ± 0.01	n.f.	0.57 ± 0.03	94.7	0.35 ± 0.01	n.f.	0.28 ± 0.01	n.f.
**Total hydroxycinnamic acids**	**0.92 ± 0.06**	**338.3**	**4.84 ± 0.23**	**155.7**	**4.11 ± 0.22**	**101.8**	**5.00 ± 0.10**	**136.7**	**3.15 ± 0.07**	**111.4**
**Flavanols**										
Epicatechin	n.d.	n.d.	n.d.	n.d.	0.02 ± 0.01	n.f.	0.01 ± 0.00	14.8	0.01 ± 0.00	2.9
Catechin	n.d.	n.d.	n.d.	n.d.	0.02 ± 0.01	68.2	n.d.	28.1	0.03 ± 0.00	36.4
Epigallocatechin	n.d.	n.d.	n.d.	n.d.	n.d.	n.d.	n.d.	n.d.	n.d.	n.d.
Gallocatechin	n.d.	n.d.	n.d.	n.d.	0.03 ± 0.00	32.0	n.d.	n.d.	n.d.	n.d.
Epicatechin-3-O-gallate	n.d.	n.d.	n.d.	n.d.	n.d.	14.0	n.d.	7.6	n.d.	0.9
Epigallocatechin-3-O-gallate	n.d.	n.d.	n.d.	n.d.	n.d.	n.d.	n.d.	n.d.	n.d.	n.d.
Epigallocatechin gallate isomer	n.d.	n.d.	n.d.	n.d.	n.d.	n.d.	n.d.	n.d.	n.d.	n.d.
Procyanidin-type B dimer isomer	n.d.	n.d.	n.d.	n.d.	n.d.	n.d.	n.d.	n.d.	n.d.	n.d.
**Total flavanols**	**n.d.**	**n.d.**	**n.d.**	**n.d.**	**0.07 ± 0.02**	**12.2**	**0.01 ± 0.00**	**5.1**	**0.05 ± 0.00**	**1.9**
**Flavanones**										
Naringenin isomer	n.d.	79.4	n.d.	3.4	0.01 ± 0.00	52.8	0.02 ± 0.00	95.1	0.04 ± 0.00	81.7
Naringenin	n.d.	n.d.	n.d.	n.d.	n.d.	n.d.	0.01 ± 0.00	23.9	0.02 ± 0.00	24.2
Tetra-hydroxyflavanone isomer	n.d.	n.d.	n.d.	n.d.	n.d.	n.d.	0.01 ± 0.00	71.0	0.02 ± 0.00	27.4
Naringenin-O-hexoside isomer 1	n.d.	n.d.	0.01 ± 0.00	n.f.	n.d.	5.8	0.04 ± 0.00	69.8	0.07 ± 0.01	46.1
Naringenin-O-hexoside isomer 2	n.d.	n.d.	0.01 ± 0.00	n.d.	0.01 ± 0.00	8.0	0.07 ± 0.00	76.9	0.11 ± 0.00	60.5
Naringenin-O-hexoside isomer 3	0.01 ± 0.00	74.6	0.01 ± 0.00	117.1	0.11 ± 0.00	34.4	0.23 ± 0.00	82.7	0.42 ± 0.01	60.9
Tetra-hydroxyflavanone-O-hexoside isomer 1	n.d.	n.d.	0.04 ± 0.01	69.1	0.19 ± 0.00	110.9	0.30 ± 0.00	102.1	0.52 ± 0.01	89.7
Tetra-hydroxyflavanone-O-hexoside isomer 2	0.01 ± 0.00	n.d.	n.d.	n.d.	0.02 ± 0.00	24.4	0.05 ± 0.00	82.4	0.06 ± 0.00	32.8
Tetra-hydroxyflavanone-O-hexoside isomer 3	n.d.	n.d.	n.d.	67.4	0.00 ± 0.00	1.5	0.01 ± 0.00	33.4	0.02 ± 0.00	13.3
**Total flavanones**	**0.03 ± 0.00**	**159.6**	**0.08 ± 0.01**	**90.3**	**0.35 ± 0.00**	**38.7**	**0.74 ± 0.00**	**83.1**	**1.29 ± 0.03**	**58.9**
**Flavones**										
Luteolin	n.d.	36.6	n.d.	53.8	n.d.	37.3	0.15 ± 0.00	85.7	0.15 ± 0.00	44.6
Tri-hydroxy-methoxyflavone isomer	n.d.	n.d.	n.d.	n.d.	n.d.	18.7	0.07 ± 0.00	65.87	0.06 ± 0.00	66.74
Apigenin-7-O-glucoside	0.01 ± 0.00	135.9	0.03 ± 0.00	108.1	0.10 ± 0.01	33.6	0.24 ± 0.00	108.1	0.62 ± 0.01	94.2
Luteolin-O-rhamnoside	n.d.	n.d.	n.d.	43.9	0.03 ± 0.00	20.5	0.23 ± 0.01	53.8	0.30 ± 0.01	52.8
Luteolin-7-O-glucoside	0.01 ± 0.00	63.5	0.02 ± 0.00	78.5	0.08 ± 0.00	n.d.	0.26 ± 0.00	105.8	0.49 ± 0.01	83.7
**Total flavones**	**0.01 ± 0.00**	**78.5**	**0.05 ± 0.00**	**84.6**	**0.21 ± 0.01**	**20.7**	**0.96 ± 0.02**	**80.5**	**1.62 ± 0.03**	**72.2**
**Flavonols**										
Quercetin	n.d.	n.d.	n.d.	n.d.	n.d.	n.d.	n.d.	0.9	n.d.	n.d.
Methyl-quercetin isomer	n.d.	n.d.	n.d.	n.d.	n.d.	n.d.	0.09 ± 0.00	72.8	0.02 ± 0.00	34.2
Isorhamnetin	n.d.	n.d.	n.d.	n.d.	n.d.	n.d.	n.d.	n.d.	n.d.	n.d.
Myricetin	n.d.	n.d.	n.d.	n.d.	n.d.	n.d.	n.d.	n.d.	n.d.	n.d.
Quercetin-3-O-pentoside	n.d.	n.d.	n.d.	n.d.	0.01 ± 0.00	4.2	0.14 ± 0.00	47.2	0.14 ± 0.00	35.0
Quercetin-3-O-pentoside isomer 1	n.d.	n.d.	n.d.	n.d.	0.01 ± 0.00	2.7	0.20 ± 0.00	51.0	0.26 ± 0.01	44.0
Quercetin-3-O-pentoside isomer 2	n.d.	n.d.	n.d.	n.d.	0.01 ± 0.00	4.8	0.21 ± 0.01	48.9	0.20 ± 0.01	31.4
Quercetin-3-O-rhamnoside	0.01 ± 0.00	72.8	0.05 ± 0.00	76.0	0.41 ± 0.02	9.8	6.93 ± 0.08	54.9	6.55 ± 0.08	34.2
Myricetin-O-rhamnoside	n.d.	n.d.	0.02 ± 0.00	21.9	n.d.	n.d.	0.48 ± 0.03	12.1	0.03 ± 0.00	1.0
Quercetin-3-O-glucoside	n.d.	n.d.	n.d.	n.d.	0.02 ± 0.00	2.5	0.43 ± 0.00	43.2	0.30 ± 0.00	15.2
Quercetin glucoside isomer	n.d.	n.d.	n.d.	n.d.	0.00 ± 0.00	n.d.	0.04 ± 0.00	83.6	0.05 ± 0.00	40.0
**Total flavonols**	**0.02 ± 0.01**	**29.3**	**0.07 ± 0.01**	**42.7**	**0.46 ± 0.02**	**6.7**	**8.53 ± 0.14**	**44.3**	**7.55 ± 0.11**	**28.4**
**Others**										
Dihydroxyphenylacetic acid isomer	0.67 ± 0.00	n.f.	1.64 ± 0.15	59.6	6.85 ± 0.21	270.3	2.38 ± 0.03	109.0	1.96 ± 0.09	129.0
Ellagic acid	15.83 ± 1.98	457.9	23.18 ± 0.75	876.7	67.67 ± 1.03	1561.5	78.08 ± 2.74	283.3	236.38 ± 5.3	1920.4
**Total others**	**16.49 ± 1.99**	**477.1**	**24.83 ± 0.89**	**459.5**	**74.53 ± 1.24**	**1085.0**	**80.46 ± 2.77**	**270.5**	**238.33 ± 5.4**	**1723.9**
**Total phenolic by MS**	**75.55 ± 4.11**	**68.6**	**81.70 ± 3.52**	**55.8**	**118.24 ± 3.72**	**53.8**	**185.95 ± 4.78**	**69.9**	**301.61 ± 7.07**	**93.9**

n.d.—compound not detected in the sample; n.f.—newly formed compound.

## Data Availability

The original contributions presented in the study are included in the article/Supplementary Material, further inquiries can be directed to the corresponding author.

## Supplementary Materials

The following supporting information can be downloaded at: https://www.mdpi.com/article/10.3390/foods13142196/s1, Table S1: Mass spectrometry data of phenolic compounds identified in the different digested samples and standards used for quantification; Table S2: Browning index of the different carob syrups; Table S3: Pearson correlation values of the different variables determined in carob syrup samples before digestion; Table S4. *p*-values from Pearson correlation of the different variables determined in carob syrup samples before digestion. Highlighted cells show a significant correlation; Table S5. Pearson correlation values of the different variables determined in carob syrup samples after in vitro digestion; Table S6. *p*-values from Pearson correlation of the different variables determined in carob syrup samples after in vitro digestion. Highlighted cells show a significant correlation.
