# Supplementary material for: Identification, Bioaccessibility, and Antioxidant Properties of Phenolic Compounds in Carob Syrup
Source: Foods. 2024 Jul 11;13(14):2196. doi: 10.3390/foods13142196 (PMC11276241; doi:10.3390/foods13142196)
Supplement: Supplementary file 1 [file foods-13-02196-s001.zip › Supplementary_materials/Table_S1.pdf]

Table S1. Mass spectrometry data of phenolic compounds identified in the different digested samples and standards used for quantification (the purity level is shown in brackets).

Compounds	m/z	Fragments	Rt	Standard
Hydroxybenzoic acid isomer 1	137.0234	93.0333	6.82	Protocatechuic acid (Sigma-Aldrich; ≥ 98%)
Hydroxybenzoic acid isomer 2	137.0234	93.0333	7.09	Protocatechuic acid (Sigma-Aldrich; ≥ 98%)
Hydroxybenzoic acid isomer 3	137.0234	93.0333	13.88	Protocatechuic acid (Sigma-Aldrich; ≥ 98%)
Dihydroxybenzoic acid isomer	153.0183	109	4.57	Protocatechuic acid (Sigma-Aldrich; ≥ 98%)
Protocatechuic acid	153.0183	109.0283; 108.0205	5.27	Protocatechuic acid (Sigma-Aldrich; ≥ 98%)
Gentisic acid	153.0183	108,0205; 109,0283	7.49	Gentisic acid (Sigma-Aldrich; ≥ 98%)
Hydroxycinnamic acid isomer 1	163.0392	119.0491; 93.0331	6.32	p-Coumaric acid (Sigma-Aldrich; ≥ 98%)
Hydroxycinnamic acid isomer 2	163.0392	119.0491; 93.0332	7.94	p-Coumaric acid (Sigma-Aldrich; ≥ 98%)
p-Coumaric acid	163.0392	119.0491; 93.0333	10.35	p-Coumaric acid (Sigma-Aldrich; ≥ 98%)
Dihydroxyphenylacetic acid isomer	167.0349	123.0441	5.66	Dihydroxyphenylacetic acid (Thermo-Fisher Scientific; ≥ 98%)
Gallic acid	169.0134	125.0233	3.13	Gallic acid (Sigma-Aldrich; ≥ 98%)
Ferulic acid	193.0506	134.0363; 178.0264; 149.0598; 93.0332	11.31	Ferulic acid (Sigma-Aldrich; ≥ 98%)
Malonyl gallic acid	255.0511	169,0135;125,0234	9.89	Gallic acid (Sigma-Aldrich; ≥ 98%)
Naringenin isomer	271.0612	119.0492; 151.0028; 107.0127; 65.0020; 177.0186; 93.0334; 83.0126; 63.0228	13.27	Naringenin (Sigma-Aldrich; ≥ 98%)
Naringenin	271.0612	119.0492; 151.0028; 107.0127; 65.0020; 177.0186; 93.0334; 83.0126; 63.0229	18.15	Naringenin (Sigma-Aldrich; ≥ 98%)
Luteolin	285.0406	133.0282; 151.0031; 175.0392; 241.0456	16.21	Apigenin (Sigma-Aldrich; ≥ 95%)
Tetra-hydroxyflavanone isomer	287.0563	125.0233; 151.0029; 135.0441	15.96	Naringin (Sigma-Aldrich; ≥ 95%)
Epicatechin	289.0721	245.0820; 109.0283; 125.0235; 205.0499	7.74	Epicatechin (Sigma-Aldrich; ≥ 98%)
Catechin	289.0721	245.0820; 109.0283; 125.0235; 205.0499	9.19	Catechin (Sigma-Aldrich; ≥ 98%)
Tri-hydroxy-methoxyflavone isomer	299.0558	284.0326; 256.0381	18.57	Apigenin (Sigma-Aldrich; ≥ 95%)
Hydroxybenzoic acid-O-hexoside isomer 1	299.0774	93.0333; 137.0234	5.76	Protocatechuic acid (Sigma-Aldrich; ≥ 98%)
Hydroxybenzoic acid-O-hexoside isomer 2	299.0774	93.0333; 137.0234	6.46	Protocatechuic acid (Sigma-Aldrich; ≥ 98%)
Ellagic acid	300.9992	283,9965; 229,0141; 145,0285; 201,0188; 173,0236; 185,0236; 257,0994	11.31	Ellagic acid (Sigma-Aldrich; ≥ 95%)
Quercetin	301.0353	151.0028; 178.9979; 121.0284; 107.0127; 65.0020; 63.0227; 83.0125; 93.0333; 169.0134; 139.0390; 117.0337	16.41	Quercetin (Sigma-Aldrich; ≥ 95%)
Epigallocatechin	305.0666	125.0233; 137.0234; 179.0338; 167.0342; 165.0184; 109.0182	5.35	Epigallocatechin (Sigma-Aldrich; ≥ 95%)

Gallocatechin	305.0666	125.0233; 137.0234; 179.0338; 167.0342; 165.0184; 109.0182	7.18	Gallocatechin (Sigma-Aldrich; ≥ 95%)
Methyl-quercetin isomer	315.0512	300.0278	17.14	Quercetin (Sigma-Aldrich; ≥ 95%)
Isorhamnetin	315.0512	300.0278	18.96	Quercetin (Sigma-Aldrich; ≥ 95%)
Dihydroxybenzoic acid-O-hexoside isomer	315.0726	109.0283; 152.0187; 153.0186	5.2	Protocatechuic acid (Sigma-Aldrich; ≥ 98%)
Myricetin	317.0302	151.0028; 137.0234; 178.9979; 109.0283; 107.0127; 65.0020; 83.0125; 289.0350; 227.0346	13.84	Myricetin (Sigma-Aldrich; ≥ 95%)
Hydroxy-methoxybenzoic acid-O-hexoside isomer	329.0880	167.0341; 152.0105; 123.0438; 108.0205	5.3	Vanillic acid (Sigma-Aldrich; ≥ 97%)
Gallic acid-O-hexoside 1	331.0674	169.0135; 125.0233	1.41	Gallic acid (Sigma-Aldrich; ≥ 98%)
Gallic acid-O-hexoside 2	331.0674	169.0135; 125.0233	2.25	Gallic acid (Sigma-Aldrich; ≥ 98%)
Gallic acid-O-hexoside 3	331.0674	169.0135; 125.0233	3.45	Gallic acid (Sigma-Aldrich; ≥ 98%)
Gallic acid-O-hexoside 4	331.0674	169.0135; 125.0233	4.07	Gallic acid (Sigma-Aldrich; ≥ 98%)
Gallic acid-O-hexoside 5	331.0674	169.0135; 125.0233	4.99	Gallic acid (Sigma-Aldrich; ≥ 98%)
Caffeoyl-hexose isomer 1	341.0881	135.0439; 179.0341; 161.0236	6.31	Caffeic acid (Sigma-Aldrich; ≥ 98%)
Caffeoyl-hexose isomer 2	341.0881	135.0439; 179.0341; 161.0236	7	Caffeic acid (Sigma-Aldrich; ≥ 98%)
Caffeoyl-hexose isomer 3	341.0881	135.0439; 179.0341; 161.0236	7.65	Caffeic acid (Sigma-Aldrich; ≥ 98%)
Gallic acid-O-glucuronide isomer 1	345.083	169,0135;125,0234	1.83	Gallic acid (Sigma-Aldrich; ≥ 98%)
Gallic acid-O-glucuronide isomer 2	345.083	169,0135;125,0234	2.18	Gallic acid (Sigma-Aldrich; ≥ 98%)
Gallic acid-O-glucuronide isomer 3	345.083	169,0135;125,0234	2.66	Gallic acid (Sigma-Aldrich; ≥ 98%)
Gallic acid-O-glucuronide isomer 4	345.083	169,0135;125,0234	3.26	Gallic acid (Sigma-Aldrich; ≥ 98%)
Ferulic acid-O-hexoside isomer 1	355.1035	193.0496; 179.0260; 149.0599; 134.0362	7.23	Ferulic acid (Sigma-Aldrich; ≥ 98%)
Ferulic acid-O-hexoside isomer 2	355.1035	193.0496; 179.0260; 149.0599; 134.0363	8.8	Ferulic acid (Sigma-Aldrich; ≥ 98%)
Ferulic acid-O-hexoside isomer 3	355.1035	193.0496; 179.0260; 149.0599; 134.0364	9.14	Ferulic acid (Sigma-Aldrich; ≥ 98%)
Ferulic acid-O-hexoside isomer 4	355.1035	193.0496; 179.0260; 149.0599; 134.0365	9.65	Ferulic acid (Sigma-Aldrich; ≥ 98%)
Syringic acid-O-hexoside isomer	359.0988	197.0450; 182.0211; 123.0078; 153.0554	5.98	Syringic acid (Sigma-Aldrich; ≥ 95%)
Dimethoxy-hydroxycinnamic acid-O-hexoside isomer	385.1144	223.0605; 208.0373; 193.0132; 179.0702	7.83	Ferulic acid (Sigma-Aldrich; ≥ 98%)

Apigenin-7-O-glucoside	431.0983	268.0381; 269.0459; 239.0351; 211.0397; 151.0028; 107.0127	13.22	Apigenin-7-O-glucoside (Extrasynthese; ≥ 98%)
Luteolin-O-rhamnoside	431.0984	285.0414; 284.0335	14.21	Luteolin-7-O-glucoside (Extrasynthese; ≥ 98%)
Quercetin-3-O-pentoside	433.0780	300.0271; 301.0356; 271.0251; 151.0029	12.19	Quercetin-3-O-glucoside (Extrasynthese; ≥ 98%)
Quercetin-3-O-pentoside isomer 1	433.0780	300.0271; 301.0356; 271.0251; 151.0031	12.67	Quercetin-3-O-glucoside (Extrasynthese; ≥ 98%)
Quercetin-3-O-pentoside isomer 2	433.0780	300.0271; 301.0356; 271.0251; 151.0030	12.42	Quercetin-3-O-glucoside (Extrasynthese; ≥ 98%)
Naringenin-O-hexoside isomer 1	433.1145	271.0614; 151.0028; 119.0490; 107.0128	11.12	Naringenin (Sigma-Aldrich; ≥ 98%)
Naringenin-O-hexoside isomer 2	433.1145	271.0614; 151.0028; 119.0490; 107.0129	11.46	Naringenin (Sigma-Aldrich; ≥ 98%)
Naringenin-O-hexoside isomer 3	433.1145	271.0614; 151.0028; 119.0490; 107.0130	13.27	Naringenin (Sigma-Aldrich; ≥ 98%)
Epicatechin-3-O-gallate	441.0827	169.0137; 125.0235; 289.0718; 245.0819	11.88	Epicatechin-3-O-gallate (Sigma-Aldrich; ≥ 98%)
Luteolin-7-O-glucoside	447.0932	285.0408; 284.0331; 227.0351; 256.0378; 151.0028; 133.0285; 107.0128; 63.0227	11.9	Luteolin-7-O-glucoside (Extrasynthese; ≥ 98%)
Quercetin-3-O-rhamnoside	447.0932	300.0280; 255.0302; 301.0359; 271.0255; 227.0349; 151.0029; 107.0128; 163.0034; 65.0020	12.93	Quercetin-3-O-rhamnoside (Sigma-Aldrich; ≥ 98%)
Tetra-hydroxyflavanone-O-hexoside isomer 1	449.1094	151.0028; 287.0564; 135.0441; 107.0127; 125.0234	9.5	Naringin (Sigma-Aldrich; ≥ 95%)
Tetra-hydroxyflavanone-O-hexoside isomer 2	449.1094	151.0027; 135.0442; 287.0562; 269.1400	11.75	Naringin (Sigma-Aldrich; ≥ 95%)
Tetra-hydroxyflavanone-O-hexoside isomer 3	449.1094	151.0028; 287.0564; 135.0441; 107.0127; 125.0234	13.71	Naringin (Sigma-Aldrich; ≥ 95%)
Epigallocatechin-3-O-gallate	457.0776	169.0134; 125.0233; 289.0718; 305.0667; 245.0819	9.49	Epigallocatechin-3-O-gallate (Sigma-Aldrich; ≥ 98%)
Epigallocatechin gallate isomer	457.0776	169.0134; 125.0233; 289.0718; 305.0667; 245.0820	9.92	Epigallocatechin-3-O-gallate (Sigma-Aldrich; ≥ 98%)
Coumaric acid-O-hexoside-pentoside	457.1357	119,0491;163,0391	8.45	p-Coumaric acid (Sigma-Aldrich; ≥ 98%)
Vanillic acid-O-hexoside-pentoside isomer 1	461.1305	167.0341; 152.0106; 123.4039; 108.0204	5.67	Vanillic acid (Sigma-Aldrich; ≥ 97%)
Vanillic acid-O-hexoside-pentoside isomer 2	461.1305	167.0341; 152.0106; 123.4039; 108.0205	6.67	Vanillic acid (Sigma-Aldrich; ≥ 97%)
Myricetin-O-rhamnoside	463.0890	317.0320	11.44	Quercetin-3-O-rhamnoside (Sigma-Aldrich; ≥ 98%)

Quercetin-3-O-glucoside	463.0882	300.0277; 301.0356; 271.0251; 255.0301; 243.0299; 151.0028; 178.9979; 107.0127	11.75	Quercetin-3-O-glucoside (Extrasynthese; ≥ 98%)
Quercetin glucoside isomer	463.0882	300.0277; 301.0356; 271.0251; 255.0301; 243.0299; 151.0028; 178.9979; 107.0128	12.4	Quercetin-3-O-glucoside (Extrasynthese; ≥ 98%)
Gallic acid-O-hexoside-O-hexoside isomer 1	493.1206	169,0135;125,0234;313,0569; 283,0463; 331,0681	4.62	Gallic acid (Sigma-Aldrich; ≥ 98%)
Gallic acid-O-hexoside-O-hexoside isomer 2	493.1206	169,0135;125,0234;313,0569; 283,0463; 331,0681	4.87	Gallic acid (Sigma-Aldrich; ≥ 98%)
Procyanidin-type B dimer isomer	577.1359	125.0234; 289.0720; 407.0791; 245.0822	7.34	Procyanidin B1 (Sigma-Aldrich; ≥ 90%)
Gallic acid-O-hexoside-O-hexoside-O-pentoside	625.163	313,0574; 125,0235; 169,0133; 493,1203;	4.92	Gallic acid (Sigma-Aldrich; ≥ 98%)
