# Supplementary material for: Identification, Bioaccessibility, and Antioxidant Properties of Phenolic Compounds in Carob Syrup
Source: Foods. 2024 Jul 11;13(14):2196. doi: 10.3390/foods13142196 (PMC11276241; doi:10.3390/foods13142196)
Supplement: Supplementary file 1 [file foods-13-02196-s001.zip › Supplementary_materials/Table_S2.pdf]

Table S2. Browning index of the different carob syrups.

Sample	K_{mix}
**C**	0.401 ± 0.02
**L**	0.214 ± 0.01
**S1**	0.255 ± 0.01
**S2**	0.146 ± 0.01
**S3**	0.100 ± 0.01
